# Astaxanthin protects against MPP^+^-induced oxidative stress in PC12 cells via the HO-1/NOX2 axis

**DOI:** 10.1186/1471-2202-13-156

**Published:** 2012-12-29

**Authors:** Qinyong Ye, Bixia Huang, Xiaodong Zhang, Yuangui Zhu, Xiaochun Chen

**Affiliations:** 1 Department of Neurology, Fujian Institute of Geriatrics, The Affiliated Union Hospital of Fujian Medical University, 29 Xinquan Road, Fuzhou, Fujian, 350001, China

**Keywords:** Parkinson’s disease, Astaxanthin, PC12 cells, MPP^+^, NOX2, HO-1

## Abstract

**Background:**

Although the etiology of PD remains unclear, increasing evidence has shown that oxidative stress plays an important role in its pathogenesis and that of other neurodegenerative disorders. NOX2, a cytochrome subunit of NOX, transports electrons across the plasma membrane to generate ROS, leading to physiological and pathological processes. Heme oxygenase-1 (HO-1) can be rapidly induced by oxidative stress and other noxious stimuli in the brain or other tissues. Astaxanthin (ATX), a carotenoid with antioxidant properties, is 100–1000 times more effective than vitamin E. The present study investigated the neuroprotective effects of ATX on MPP^+^-induced oxidative stress in PC12 cells.

**Results:**

MPP^+^ significantly decreased MTT levels in a concentration-dependent manner. Hemin, SnPPIX and ATX didn’t exhibit any cytotoxic effects on PC12 cells. Pretreatment with ATX (5, 10, 20 μM), caused intracellular ROS production in the MPP^+^ group to decrease by 13.06%, 22.13%, and 27.86%, respectively. MPP^+^ increased NOX2, NRF2 and HO-1 protein expression compared with control (p < 0.05). Co-treatment with hemin or ATX suppressed NOX2 expression (p < 0.01), and greatly increased NRF2 and HO-1 expression (p < 0.01). MPP^+^ treatment up-regulated both NOX2 (p < 0.01) and HO-1 (p < 0.01) mRNA levels. Co-treatment with hemin or ATX significantly increased HO-1 mRNA levels (p < 0.01), and decreased NOX2 mRNA levels (p < 0.01). MPP^+^ increased NOX2 and HO-1 expression with considerable fluorescence extending out from the perinuclear region toward the periphery; this was attenuated by DPI. Co-treatment with hemin or ATX significantly up-regulated HO-1 expression and decreased NOX2 expression with considerable fluorescence intensity (stronger than the control and MPP^+^ groups).

**Conclusions:**

ATX suppresses MPP^+^-induced oxidative stress in PC12 cells via the HO-1/NOX2 axis. ATX should be strongly considered as a potential neuroprotectant and adjuvant therapy for patients with Parkinson’s disease.

## Background

Parkinson’s disease (PD) is a progressive, neurodegenerative movement disorder, characterized by the loss of nigrostriatal dopaminergic neurons. Although the etiology of PD remains unclear, increasing evidence has shown that oxidative stress plays an important role in its pathogenesis [1] and that of other neurodegenerative disorders. Several mechanisms have been proposed to explain the pathogenesis of PD, including the production of reactive oxygen species (ROS), generated by dopamine auto-oxidation, mitochondrial dysfunction, or α-synuclein deposition [2,3]. Excessive accumulation of ROS, an imbalance of antioxidant enzymes and activation of the oxidase system all damage DNA and induce lipid peroxidation and protein modification, subsequently causing cellular dysfunction and even apoptosis [4,5]. PC12 cells, derived from a clonal rat pheochromocytoma cell line, have been widely used as cellular models of Parkinson’s disease as these cells share features with midbrain dopaminergic neurons [6,7]. The neurotoxin 1-methyl-4-phenyl-1,2,3,6-tetrahydropyridine (MPTP), via its active metabolite 1-methyl-4-phenylpyridinium ion (MPP^+^), is selectively taken up by dopaminergic neurons via the plasma membrane dopamine transporter [8] impairing dopamine uptake and eliciting dopaminergic neuronal loss. Further understanding into the mechanisms of MPP^+^-induced dopaminergic neuronal death may provide insights into potential therapeutic targets for PD.

The production of ROS by mitochondria is thought to be the main cause of oxidative stress. However, a role for the ROS-generating nicotinamide adenine dinucleotide phosphate (NADPH) oxidase (NOX) enzymes has recently emerged. NADPH oxidase plays a critical role in CD200-CD200R-mediated dopamine neurotoxicity in PD [9]. NOX2 is a cytochrome subunit of NOX that transports electrons across the plasma membrane to generate ROS and promotes physiological and pathological processes. Studies have shown NOX2 expression in several areas of the adult brain, including the corpus callosum, spinal cord, hippocampus, cerebral cortex, brainstem, amygdala, striatum, thalamus, cerebellum, etc. [10]. Activation of phagocytic NOX2 has been studied mainly in microglia, where it plays a role in inflammation, but may also contribute to neuronal death in pathological conditions [10]. Thus, microglia-mediated NOX2 activation, caused by dopaminergic neuron injury, may play a role in the loss of dopaminergic neurons.

Heme oxygenase-1 (HO-1), a 32-kDa cellular stress response protein (also known as Hsp32), can be rapidly induced by oxidative stress and other noxious stimuli in the brain or other tissues [11]. HO-1 is the rate-limiting enzyme in heme degradation and is therefore involved in the control of cellular heme content. HO-1 has many anti-oxidant properties, giving the enzyme protective properties in various models of oxidative injury. Indeed, HO-1 over-expressing mice have decreased oxidative damage [12]. Increased HO-1 expression has a cytoprotective effect against MPP^+^-induced cytotoxicity [13]. NRF2 (NF-E2-related factor) is a transcription factor that induces the expression of various cytoprotective enzymes. NRF2 activation and subsequent cytoprotective gene induction promote the restoration of balance between oxidants and antioxidants after an oxidative insult. Studies have reported that NRF2 can regulate HO-1 protein or mRNA levels. The coffee diterpene kahweol induces HO-1 via the PI3K and p38/NRF2 pathways to protect human dopaminergic neurons from 6-hydroxydopamine (6-OHDA)-induced oxidative stress [14]. Transfection with NRF2 siRNA significantly suppressed cigarette smoke particle-phase extract (CSPE)-enhanced HO-1 protein levels. Activated NRF2 is recruited to the promoter region of HO-1, leading to increased expression of HO-1 protein in human tracheal smooth muscle cells (HTSMCs) [15]. Up-regulation of HO-1 by activation of the NRF2-ARE signaling pathway attenuates paraquat-mediated oxidative stress and cell death in dopaminergic PC12 cells [16].

**Figure 1:**
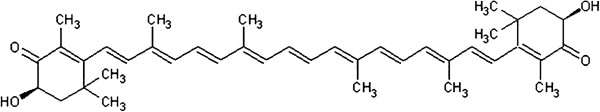
Chemical structure of Astaxanthin.

Astaxanthin (3,3^′^-dihydroxy-β,β-carotene-4,4^′^-dione; ATX; Figure 1), a carotenoid with antioxidant properties, is 100–1000 times more effective than vitamin E. ATX is commonly found in crustaceans such as shrimp and crab, as well as marine organisms such as salmon, salmon roe, krill and algae. ATX is found in the free state and in the ester form and also exists as a chromoprotein. In recent years, a number of *in vitro* and *in vivo* studies of ATX have demonstrated its antioxidant and neuroprotective effects, for example in global cerebral ischemia in rats [17]. In addition, ATX has been shown to inhibit 6-OHDA-induced neuronal apoptosis [18] and protect PC12 cells against beta-amyloid peptide 25–35-induced cell apoptosis and death [19]. A study using rats fed natural ATX revealed ATX crossed the blood–brain barrier in mammals, extending its antioxidant benefits into the brain [20]. However, the neuroprotective action of ATX in PD has yet to be investigated.

The present study investigated the neuroprotective effects of ATX on MPP^+^-induced oxidative stress in PC12 cells.

## Methods

### Materials

Dulbecco’s modified Eagle’s medium (DMEM), fetal bovine serum (FBS), Hank’s balanced salt solution (HBSS) and antibiotic-antimycotic were purchased from Gibco BRL (Grand Island, NY, USA), astaxanthin (ATX) from Wako (Catalog No. 013–23051, Tokyo, Japan), N-methyl-4-phenylpyridinium (MPP^+^) ion (No. D048), the NADPH oxidase inhibitor diphenyleneiodonium chloride (DPI, No. D2926), the HO inducer hemin (ferriprotoporphyrin IX chloride, No. 51280) and 3-[4,5-dimethylthiazol- 2-yl]-2,5- diphenyltetrazolium bromide (MTT) from Sigma-Aldrich (St. Louis, MO, USA), the HO inhibitor tin protoporphyrin IX dichloride (SnPPIX, Cat. No. 0747) from Tocris Bioscience (Abingdon, UK), 4^′^,6-diamidino-2-phenylindole (DAPI) and 2^′^,7^′^-dichlorfluorescein-diacetate (DCFH-DA) from Beyotime Institute of Biotechnology (Shanghai, China), All other chemicals were purchased from commercial sources.

### Cell culture

The rat pheochromocytoma cell line (PC12) was cultured in high glucose DMEM, supplemented with 10% FBS, 100 U/ml penicillin, and 100 U/ml streptomycin. The cell line was grown as undifferentiated cells in a 100-mm^2^ culture dish at 37°C in a humidified incubator (Forma Scientific, Ohio, USA; Model No. 3130) containing 5% CO2. When the cells were 70% confluent they were harvested and dispersed. The well dispersed cells were then cultured for 24–36 h with an antagonist or ATX in the presence or absence of MPP^+^. The cultured medium was changed every 2–3 d. In some experiments, cells were pre-treated for 2 h with 20 μM hemin, 10 μM SnPPIX, 10 μM ATX and 1 μM DPI, and stimulated with MPP^+^ (500 μM) for 24 h. Control cells were cultured without MPP^+^.

### Cell viability assay

MTT, absorbed into the cell and eventually the mitochondria, is broken down into formazan by mitochondria succinate dehydrogenase. Accumulation of formazan reflects the activity of mitochondria directly and the cell viability indirectly. Cell viability was measured by the MTT assay. PC12 cells were seeded on 96-well plates at a density of 8×10^3^ cells/well, cultured, differentiated, and treated according to the above methods. A total of 20 μl of MTT was added at a concentration of 0.5 mg/ml after media (200 μl) was added to each well. The plates were incubated at 37°C for 4 h to dissolve the formazan that had formed. The solution (220 μl) was removed from each well and 150 μl of dimethyl sulfoxide was added. Reduced MTT was measured on an ELISA reader (Bio-Rad, Hercules, CA, USA) at a wavelength of 570 nm. Values for each treatment group are expressed as a percentage of the control value.

### Detection of intracellular ROS

The DCFH-DA assay was used to measure ROS production in differentiated PC12 cells treated with MPP^+^. DCFH-DA is a fluorescent dye that crosses the cell membrane and is enzymatically hydrolyzed by intracellular esterases to non-fluorescent DCFH. The cells were plated at a density of 4×10^5^ cells per 6-well dish. Differentiated PC12 cells were pretreated with DPI (1 μM) and ATX (5, 10, 20 μM) in medium for 2 h, then exposed to MPP^+^ (500μM) for 24 h. The cells were incubated with DCFH-DA at a final concentration of 10 μM in high glucose DMEM without FBS for 20 min at 37°C and washed three times with DMEM. ROS levels were measured using a flow cytometer (FACScalibur, Becton Dickinson, San Jose, CA, USA) with excitation and emission wavelengths set at 475 and 525 nm, respectively. For each analysis, 10,000 events were recorded. The value for each treatment group was converted to a percentage of the control value.

### Western blot analysis

The cells, plated at a density of 4×10^5^ cells per 6-well dish, were treated with various concentrations of antagonist or ATX in media with or without MPP^+^ (500μM) for 24 h. For whole cell lysates, the cells were washed twice with ice cold PBS, harvested in RIPA lysis buffer (50 mM Tris pH 7.4, 150 mM NaCl, 1% Triton X-100, 1% sodium deoxycholate, 0.1% SDS, sodium orthovanadate, sodium fluoride, EDTA, 0.5 mM PMSF), incubated for 10 min on ice, centrifuged at 12,000 × *g* for 10 min at 4°C and the supernatant, containing cell lysates, collected. Equal amounts of protein (50 μg) from the cell extracts in each treatment condition were separated using 10% sodium dodecyl sulfate (SDS) polyacrylamide gel electrophoresis (PGE), then transferred electrophoretically onto polyvinylidene fluoride (PVDF) (Millipore, Carrigtwohill, Ireland). The blots were blocked by incubation in 5% (w/v) non-fat dry milk in PBS with 0.1% Tween 20 (PBS-T) for 4 h. After incubation with a primary antibody [anti-NOX2 (Santa Cruz Biotechnology, Santa Cruz, CA, USA) 1:200 and anti-HO-1 (Stressgen, Ann Arbor, MI, USA) 1:1000] in PBS-T at 4°C overnight, the membranes were washed three times in PBS-T for 10 min. Subsequently, the membranes were incubated for 1 h in PBS-T containing the appropriate horseradish peroxidase-conjugated secondary antibody [anti-mouse IgG and anti-rabbit IgG (Beyotime Institute of Biotechnology, China) 1:2000]. The immunoreactive bands were visualized and quantified using the Luminata Forte Western HRP substrate (Millipore, Billerica, MA, USA). Protein levels were normalized to the housekeeping protein β-actin (Beyotime Institute of Biotechnology, Shanghai, China) 1:1000to adjust for variability of protein loading and expressed as a percentage of the vehicle control.

### Immunofluorescence confocal microscopy

PC12 cells were permeabilized and fixed with 4% paraformaldehyde and 0.5% Triton X-100. Slides were blocked with 1% normal donkey serum (Merck, Darmstadt, Germany) in PBS for 30 min at room temperature. Cells were washed with 0.1% BSA (Beyotime Institute of Biotechnology, Shanghai, China)/PBS three times with gentle shaking, then incubated with the primary antibodies diluted (HO-1 1:1000 and NOX2 1:200) in 0.1% BSA/PBS at 4°C overnight. Labeled donkey anti–rabbit IgG or anti-mouse IgG (Invitrogen, Paisley, UK) (1:1000 dilution) were used as the secondary antibody and incubated in the dark for 2 h at room temperature. Specific antibody binding was detected by Alexa Fluor 488- (green label) and Alexa Fluor 594- (red label) conjugated extravidin (Sigma-Aldrich,St. Louis, MO, USA). Confocal microscopy was performed using the Leica SP5 confocal microscopy system (Leica Microsystems CMS GmbH, Mannheim, Germany). Optical sections were taken at 0.5 μm intervals and images were captured and stored digitally for analysis. Fluorescence intensity was quantified from at least three random fields (1024 × 1024 pixels; 310 × 310 μm) per slide, three slides per experimental condition, and repeated three times using separate cell cultures.

### Quantitative real-time PCR analysis

Total RNA from PC12 cells was isolated according to the manufacturer’s protocol using TRIzol reagent (Invitrogen, Carlsbad, CA, USA). Total RNA purity and integrity was confirmed using the ND-1000 NanoDrop (NanoDrop Technologies, Wilmington, USA) and 2100 Bioanalyzer (Agilent, California, USA). RNA (1 μg) was reverse-transcribed into cDNA in a total volume of 20 μl using the RevertAid^TM^ First Strand cDNA Synthesis Kit (Fermentas, St. Leon-Rot, Germany). The cDNA (2 μl) was amplified with a sequence detection system (ABI Prism 7500) in a total volume of 20 μl containing 10 μl of the FastStart Universal SYBR Green Master Mix (ROX) (Roche, Penzberg, Germany) and each primer at 0.3 μM. Forward and reverse primers for spe-cific amplification of HO-1 [F1(5^′^-CAAGCAGAACCCAGTCTATGC-3^′^) and [R1(5^′^-GATGAGTACCTCCCACCTCGT-3^′^)], NOX2 [F1(5^′^-CTGCCTCCATTCTCAAGTCTG-3^′^) and [R1(5^′^-ATTCATCCCAGCCAGTAAGGT-3^′^)] and β-actin [F1(5^′^-CACCCGCGAGTACAACCTTC-3^′^) and [R1(5^′-^ CCCATACCCACCATCACACC-3^′^)] were designed, eliminating the possibility of amplifying genomic DNA. Quantitative real-time PCR was performed using the ABI prism 7500 HT sequence detection system (Applied Biosystems, Foster City, CA, USA) based on the 59-nuclease assay [21] for the various genes indicated and the housekeeping gene GAPDH. Relative expression was calculated using the ΔΔCt method [22], and passed the validation experiment. The results are expressed as an average of triplicate samples of at least three independent experiments for control and treated cells.

### Statistical analysis

All statistical analyses were carried out using one-way ANOVAs with repeated measures followed by Scheffe’s *post hoc* tests. A p value below 0.05 was deemed statistically significant.

## Results

### Effects of MPP^+^, hemin , SnPPIX, DPI and ATX on cell viability

**Figure 2:**
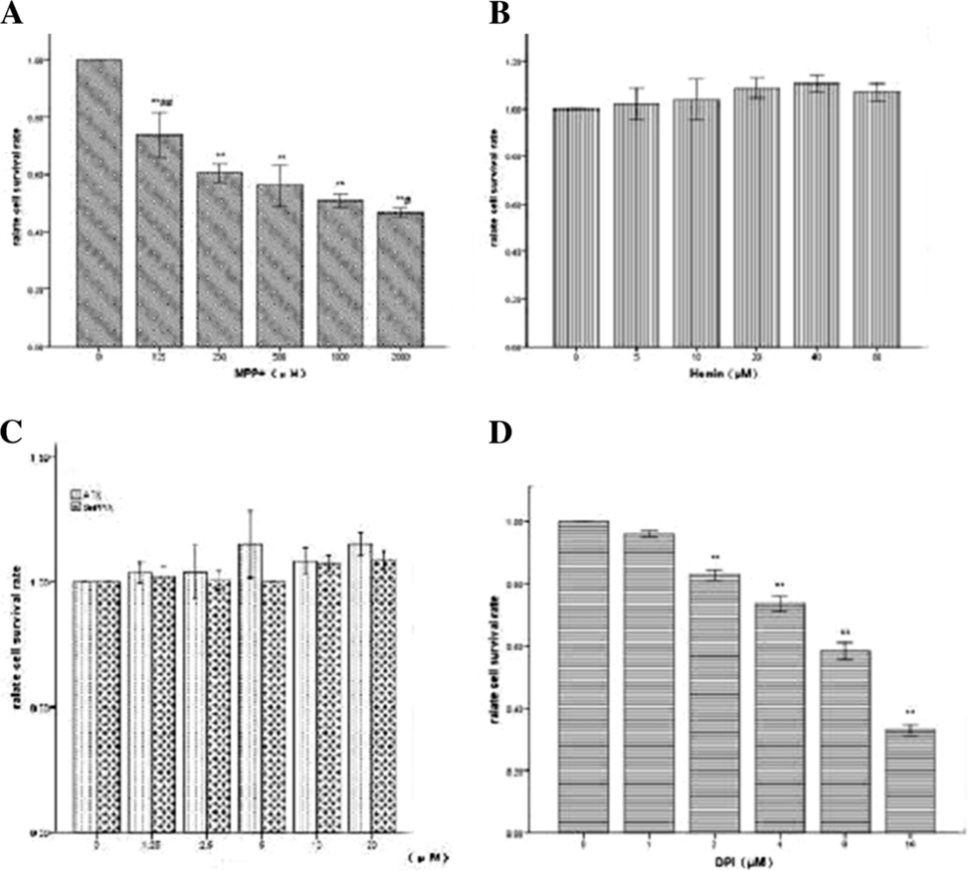
**Effects of MPP**^**+**^**, hemin, SnPPIX, DPI and ATX on cell viability.** Cells were treated with different concentrations of MPP^+^, hemin, SnPPIX, DPI and ATX for 24 h, after which the MTT assay was performed. (**A**) MPP^+^ (0–2000 μΜ); (**B**) Hemin (0–40 μΜ); (**C**) SnPPIX and ATX (0–20 μΜ); (**D**) DPI (0–16 μΜ). Relative cell survival values are expressed as a percentage of the control value, *p < 0.05 vs. untreated control, **p < 0.01 vs. untreated control, # p < 0.05 vs. MPP^+^ (500 μΜ) treatment only, ## p < 0.01 vs. MPP^+^ (500 μΜ) treatment only.

To evaluate the viability of differentiated PC12 cells after exposure to oxidative injury, differentiated PC12 cells were treated with different concentrations of MPP^+^ (125–2000 μmoL/L) for 24 h. After washing, and following 24 h incubation, cell viability was measured using the MTT assay. MPP^+^ significantly decreased MTT levels, indicating a lower cell count, in a concentration-dependent manner (Figure 2A). Treatment with a concentration of 500μΜ of MPP^+^ for 24 h was selected for subsequent experiments as it reduced cell viability to approximately 44% of control (p < 0.01, Figure 2A). Next, the effects of hemin, SnPPIX, DPI and ATX on cell viability were investigated. Hemin didn’t exhibit any cytotoxic effects on PC12 cells at concentrations ranging from 5–40 μΜ (p > 0.05, Figure 2B), while SnPPIX and ATX did not significantly alter cell viability at concentrations ranging from 1.25–20 μΜ (p > 0.05, Figure 2C). For experimental testing, a concentration of 20 μΜ for hemin, 10 μΜ for SnPPIX and 10 μΜ ATX were selected. The most commonly used NOX inhibitor is DPI. At low concentrations (1 μΜ), DPI attenuates the viability of differentiated PC12 cells, while at higher concentrations (1–16 μΜ) the effect of DPI on cell viability is more significant (Figure 2D). A concentration of 1 μΜ, which reduced cell viability by approximately 4.2% compared with control (p = 0.075, Figure 2D), was selected to block expression of NOX2.

### Effect of ATX on MPP^+^-induced oxidative stress

**Figure 3:**
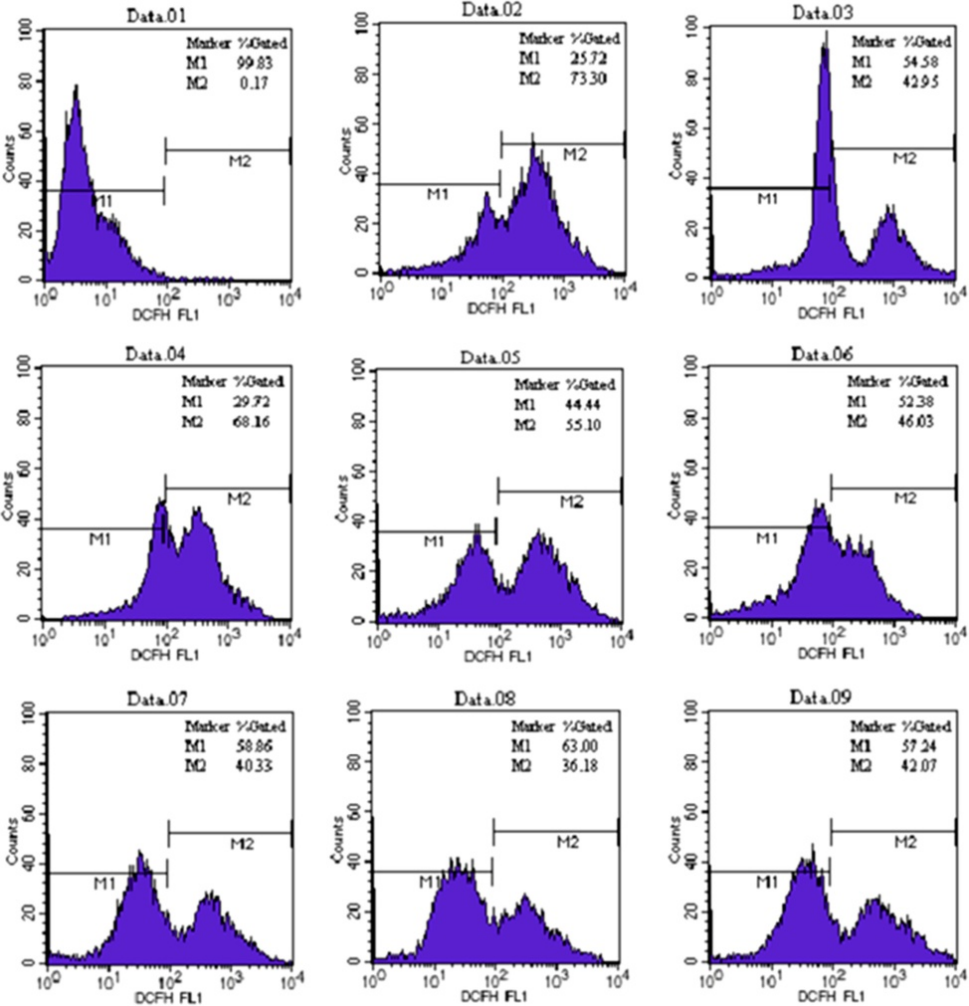
**ATX attenuates the generation of intracellular ROS by MPP^+^-induced oxidative stress in PC12 cells.** PC12 cells treated groups: Data.01: Negative control without DCFH-DA; Data.02: Positive control with Rosup; Data.03: control; Data.04: MPP^+^ (500μM); Data.05: MPP^+^ (500μM) and ATX (5μM); Data.06: MPP^+^ (500μM) and ATX (10μM); Data.07: MPP^+^ (500μM) and ATX (20μM); Data.08: ATX (10μM); Data.09: MPP^+^ (500μM) and DPI (1μM). Intracellular ROS generation was determined by DCFH-DA fluorescence.

The events involved in MPP^+^-induced oxidative stress were evaluated to determine the amount of intracellular ROS production and the effect of ATX on ROS production. ROS production was measured using DCFH-DA. As shown in Figure 3, the results show MPP^+^ increased ROS production by 25.21%, which was inhibited by DPI. Pretreatment with 5, 10 and 20 μM ATX caused intracellular ROS production in the MPP^+^ group to decrease by 13.06%, 22.13% and 27.86%, respectively. ATX alone attenuated ROS production by 6.77%.

### Effects of hemin, SnPPIX and DPI on MPP^+^-induced NOX2, NRF2 and HO-1 levels in PC12 cells

**Figure 4:**
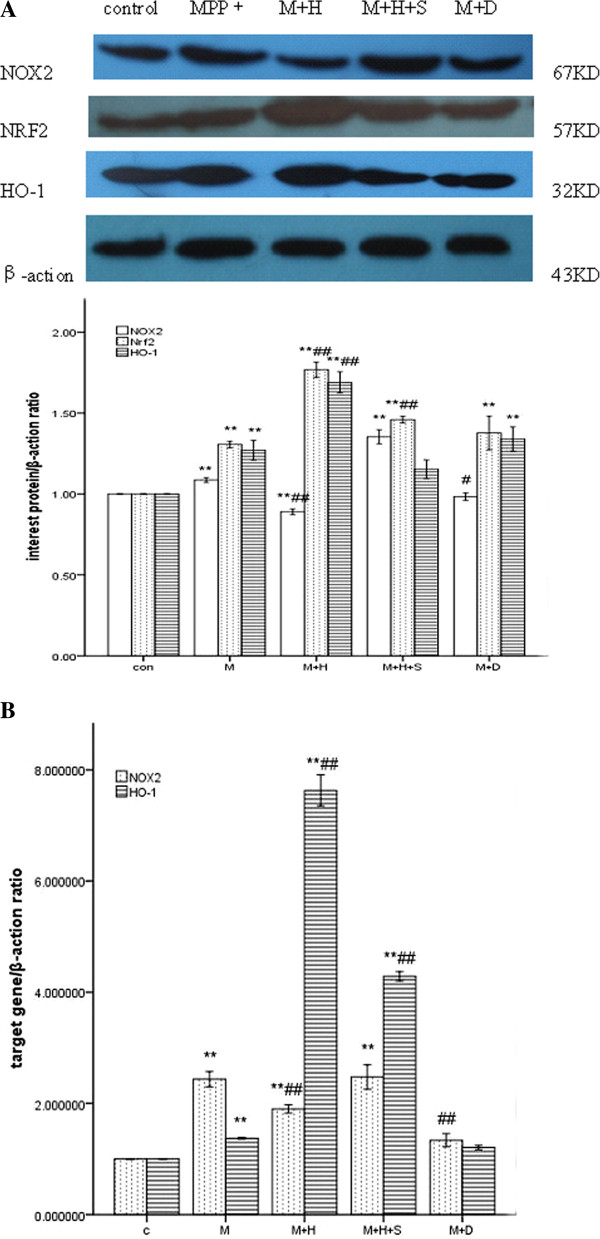
**Effects of hemin, SnPPIX and DPI on MPP^+^-induced NOX2, NRF2 and HO-1 levels in PC12 cells.** The groups are C: Control; M: MPP^+^ (500 μM); M+H: MPP^+^ (500 μM) plus hemin (20 μM); MHS: MPP^+^ (500 μM) plus hemin (20 μM) and SnPPIX (10 μM); MD: MPP^+^ (500 μM) plus DPI (1 μM). (**A**). The protein expression of NOX2, NRF2 and HO-1 was determined by western blot analysis; (**B**). NOX2 and HO-1 mRNA levels were determined by quantitative real-time PCR; (**C**).

**Figure 5:**
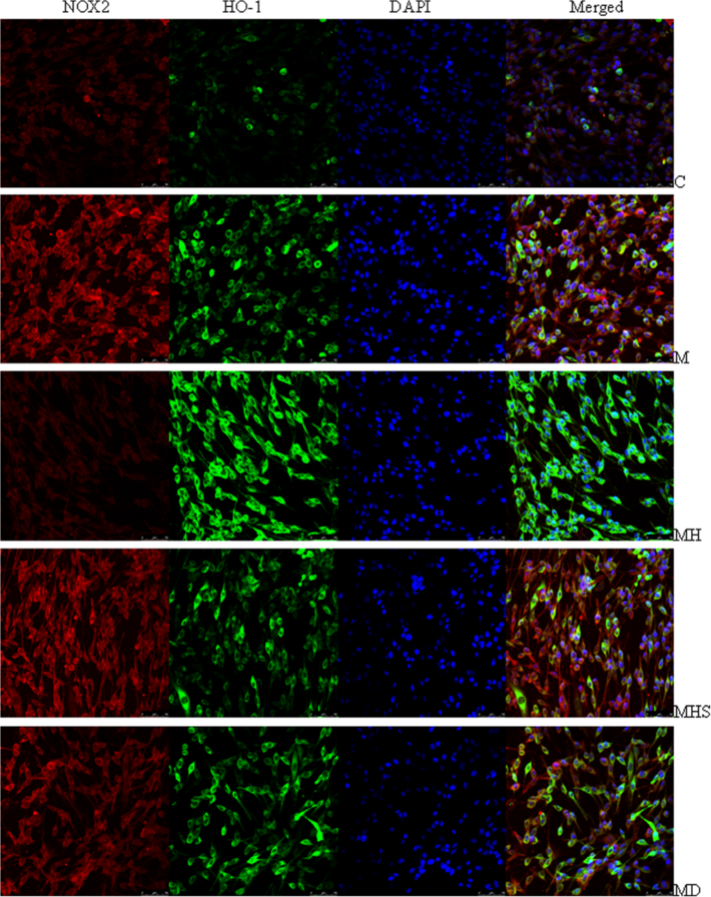
**The subcellular localization and expression of HO-1 and NOX2 following administration of MPP^+^ and hemin.** The groups are C: Control; M: MPP^+^ (500 μM); M+H: MPP^+^ (500 μM) plus hemin (20 μM); MHS: MPP^+^ (500 μM) plus hemin (20 μM) and SnPPIX (10 μM); MD: MPP^+^ (500 μM) plus DPI (1 μM). Confocal microscopic images of the subcellular localization and expression of HO-1 and NOX2. After the administration of MPP^+^ and other reagents to PC12 cells, NOX2 (Alexa Fluor 594, red fluorescence), HO-1 (Alexa Fluor 488, green fluorescence), and nuclei counterstained with DAPI (blue) are shown.

Differentiated PC12 cells were pretreated with hemin, SnPPIX and DPI in medium for 2 h and exposed to MPP^+^ (500 μM) for 24 h. To determine the relationship between NOX2 and HO-1 expression, PC12 cells were treated with control, MPP^+^ (500 μM), MPP^+^ (500 μM) plus hemin (20 μM), MPP^+^ (500 μM) plus hemin (20 μM) and SnPPIX (10 μM), and MPP^+^ (500 μM) plus DPI (1 μM). Protein expression of NOX2, NRF2 and HO-1 was determined by western blot analysis. As shown in Figure 4A, MPP^+^ increased NOX2 expression compared with control (p < 0.05, Figure 4A and Figure 5A). Co-treatment with hemin suppressed NOX2 expression compared with control (p < 0.01). Co-treatment with hemin and SnPPIX increased NOX2 expression compared with control (p < 0.01). Finally, co-treatment with the NADPH inhibitor DPI decreased NOX2 expression compared with the MPP^+^ treated group (p < 0.05). Increased NRF2 and HO-1 expression is shown following MPP^+^ treatment for 24 h compared with control (p < 0.01), indicating that MPP^+^ enhanced NOX2, NRF2 and HO-1 expression in PC12 cells. Co-treatment with hemin greatly increased HO-1 expression compared with control (100–168.9%) (p < 0.01, Figure 4A), Finally, although co-treatment with SnPPIX enhanced HO-1 expression, the effect was not significant (p = 0.091, Figure 4A).

To determine whether the relationship between NOX2 and HO-1 expression occurred at the transcriptional level, NOX2 and HO-1 mRNA expression was determined using quantitative real-time PCR. Our results show that MPP^+^ treatment up-regulated both NOX2 (from 100% control to 243.5%, p < 0.01, Figure 4B) and HO-1 mRNA (from 100% control to 137.1%, p < 0.01, Figure 4B) levels. Co-treatment with hemin significantly increased HO-1 mRNA levels by 663% vs. control and 626% vs. the MPP^+^ group (p < 0.01, Figure 4B), and decreased NOX2 mRNA levels by 53.5% vs. the MPP^+^ group (p < 0.01, Figure 3B). Co-treatment with hemin and SnPPIX decreased HO-1 mRNA levels by 335% vs. the MPP^+^ plus hemin group and increased NOX2 expression by 58% vs. the MPP^+^ plus hemin group (p < 0.01, Figure 4B). Finally, co-treatment with DPI did not alter NOX2 mRNA levels compared with control (p = 0.106). However, DPI treatment decreased NOX2 mRNA levels by 110% compared with the MPP^+^ group (p < 0.01, Figure 4B).

We then performed immunofluorescent double staining to examine the subcellular localization and expression of HO-1 and NOX2, following administration of MPP^+^ and other reagents (Figure 5). Under normal growth conditions, NOX2 and HO-1 were detected in the perinuclear region. MPP^+^ treatment increased NOX2 expression, with significant fluorescence extending out toward the peri-phery, which was attenuated by DPI (Figure 5). Hemin significantly upregulated HO-1 expression, with stronger fluorescence intensity compared with both the control and MPP^+^ groups, and significantly decreased NOX2 expression. Both hemin-induced effects were attenuated by SnPPIX treatment (Figure 5). The results indicate that MPP^+^ treatment upregulated NOX2, NRF2 and HO-1 expression at both the mRNA and protein levels.

### Effects of ATX on MPP^+^-induced changes in NOX2, NRF2 and HO-1 levels in PC12 cells

**Figure 6:**
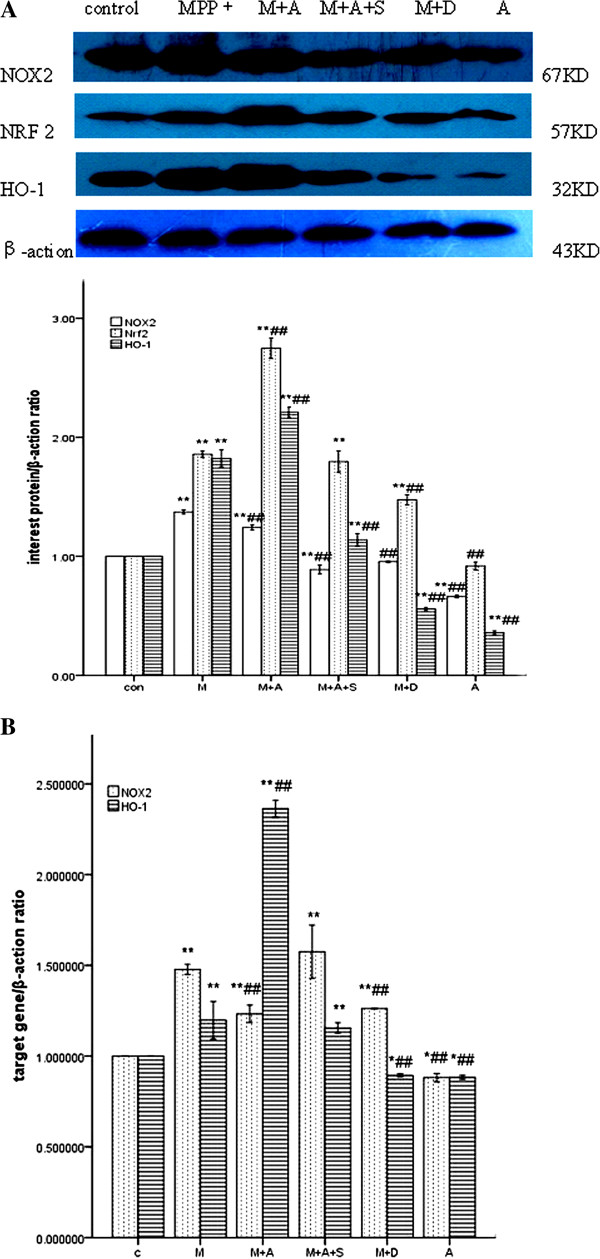
**Effects of ATX on MPP^+^-induced NOX2, NRF2 and HO-1 levels in PC12 cells.** The groups are C: Control; M: MPP^+^ (500 μM); M+A: MPP^+^ (500 μM) plus ATX (10 μM); MAS: MPP^+^ (500 μM) plus ATX (10 μM) and SnPPIX (10 μM); MD: MPP^+^ (500 μM) plus DPI (1 μM), A: ATX (10 μM). (**A**). The protein expression of Nox2, NRF2 and HO-1 was determined by western blot analysis; (**B**). NOX2 and HO-1 mRNA levels were determined by quantitative real-time PCR.

Previous findings have shown that ATX treatment alters the expression of the antioxidant enzyme HO-1, protects neurons against Aβ-induced cytotoxicity [23], and protects against oxygen-glucose deprivation-induced oxidative stress-dependent injury [18]. We first investigated the possibility that ATX might alter the expression of NOX2. Differentiated PC12 cells were pretreated with ATX, SnPPIX and DPI in the medium for 2 h then exposed to MPP^+^ (500 μM) for 24 h. To determine whether ATX enhances HO-1 expression and inhibits NOX2 expression to protect against MPP^+^-mediated cytotoxicity in PC12 cells, cells were treated with control, MPP^+^ (500 μM), MPP^+^ (500 μM) plus ATX (10 μM), MPP^+^ (500 μM) plus ATX (10 μM) and SnPPIX (10 μM), MPP^+^ (500 μM) plus DPI (1 μM), and ATX(10 μM). As shown in Figure 6A, MPP^+^ increased NOX2 expression compared with control (p < 0.05, Figure 3A and Figure 6A). Co-treatment with ATX decreased NOX2 expression compared with the MPP^+^ group (p < 0.01), while co-treatment with the NADPH inhibitor DPI significantly decreased NOX2 expression compared with the MPP^+^ group (p < 0.01). MPP^+^ (500 μM) treatment of PC12 cells for 24 h increased NRF2 and HO-1 expression compared with control (p < 0.01). Co-treatment with ATX significantly increased both HO-1 (100–221.5%) and NRF2 expression (100–274.8%) compared with control (p < 0.01, Figure 6A). These increases were attenuated by SnPPIX. Finally, ATX treatment alone did not alter NRF2 expression (p = 0.536, Figure 6A).

To examine whether ATX affects NOX2 and HO-1 expression at the transcriptional level, NOX2 and HO-1 mRNA expression was determined by quantitative real-time PCR. It revealed that co-treatment with MPP^+^ and ATX significantly increased HO-1 mRNA levels by 117% vs. the MPP^+^ group (p < 0.01, Figure 6B) and decreased NOX2 mRNA levels by 24.4% vs. the MPP^+^ group (p < 0.01, Figure 6B). Co-treatment with MPP^+^, ATX and SnPPIX decreased HO-1 mRNA levels by 121% vs. the MPP^+^ plus ATX group and increased NOX2 expression by 34% vs. the MPP^+^ plus ATX group (p < 0.01, Figure 6B). Co-treatment with DPI decreased NOX2 mRNA levels compared with the MPP^+^ group (p < 0.01, Figure 6B). Finally, ATX treatment alone decreased NOX2 and HO-1 mRNA levels by 11.8% compared with control (p < 0.01, Figure 6B).

**Figure 7:**
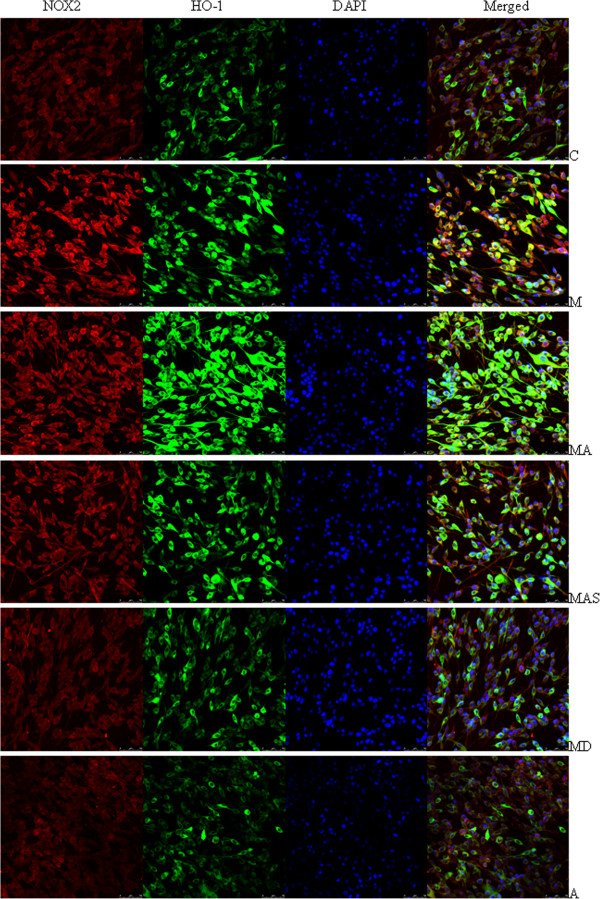
**The subcellular localization and expression of HO-1 and NOX2 following administration of MPP^+^ and ATX.** The groups are C: Control; M: MPP^+^ (500 μM); M+A: MPP^+^ (500 μM) plus ATX (10 μM); MAS: MPP^+^ (500 μM) plus ATX (10 μM) and SnPPIX (10 μM); MD: MPP^+^ (500 μM) plus DPI (1 μM), A: ATX (10 μM). Confocal microscopic images of the subcellular localization and expression of HO-1 and NOX2. After administration of MPP^+^ and other reagents to PC12 cells, NOX2 (Alexa Fluor 594, red fluorescence), HO-1 (Alexa Fluor 488, green fluorescence), and nuclei counterstained with DAPI (blue) are shown.

Finally, we analyzed confocal microscopic images of HO-1 and NOX2 subcellular localization and expression after treatment with MPP^+^ and other reagents (Figure 7). In MPP^+^-induced PC12 cells, MPP^+^ increased NOX2 expression with considerable fluorescence extending out from the perinuclear region toward the periphery, which was attenuated by DPI (Figure 7). MPP^+^ also increased HO-1 expression. Co-treatment with ATX signifi-cantly up-regulated HO-1 expression, with considerable fluorescence intensity (stronger than the control and MPP^+^ groups), which was attenuated by SnPPIX. Finally, co-treatment with ATX significantly decreased NOX2 expression (Figure 7).

## Discussion

There is increasing evidence that oxidative stress plays a key role in the pathogenesis of PD. The present study was designed to elucidate the potential molecular mechanisms and antioxidant effects of ATX, a natural carotenoid found in aquatic animals, in an *in vitro* model of PD. We tested the protective effects of ATX on MPP^+^-induced cytotoxicity. We found that ATX pretreatment significantly reduced oxidative stress in PC12 cells treated with MPP^+^.

NOX2 plays a key role in microglia-mediated dopaminergic neurotoxicity, such as exposure to rotenone, LPS, MPTP/MPP^+^, 6-OHDA and angiotensin II, which induces TH-positive selective neuronal death in the midbrain [24]. NOX2-deficient mice are protected against dopaminergic neurotoxicity in an MPTP-induced PD mouse model. Dopamine degeneration decreased by 20% in MPTP-treated NOX2−/− mice compared with the wild-type control group [25]. NOX2 is highly relevant to nervous system diseases, as it regulates the growth, proliferation, activation and apoptosis of neurons, making NOX2 suppression a promising therapeutic strategy. In our study, MPP^+^ significantly increased the level of NOX2 expression, which was subsequently attenuated by DPI (Figure 4 and Figure 6). However, DPI is not a specific inhibitor of NOX but acts on all flavoproteins.

Induction of HO-1 inhibits NOX activity in macrophages [26], although the mechanism by which HO-1 modulates NOX activity is not totally clear. The inhibitory effect of HO-1 has been observed in both the aorta and kidney, tissues in which NOX activation is a major source of oxidative stress [27]. Hemin decreases cardiac oxidative stress, involving PI3Kinase/Akt pathway signaling regulation via HO-1/BVR over-expression and modifying NOX activation [28]. In this study, we provide direct evidence that HO-1 modulates NOX2 activity. Hemin, as an HO-1 inducer, can increase HO-1 and NRF2 levels. Our study found that hemin significantly decreased NOX2 activity compared with the MPP^+^ group; this decrease was subsequently attenuated by SnPPIX treatment (Figure 4, 5). The present study suggests that over-expression of HO-1 limits oxidative damage mediated by NOX2 activation in MPP^+^-treated PC12 cells. This is an important mechanism underlying the neuroprotective effects of HO-1 and NRF2, proposed by several *in vitro* and *in vivo* studies. Studies have shown that up-regulation of HO-1 expression and the subsequent increase in HO activity may confer an adaptive neuroprotective response to oxidative insults both *in vitro* and *in vivo*, mediated by the activation of NRF2 [29,30]. Indeed, in a study using the acute MPTP model, it was shown that NRF2−/− mice are more sensitive to MPTP [31].

ROS have multiple effects on cell function, depending on the amount and subcellular location of the ROS generated. Controlled intracellular ROS production from NOX is necessary for normal cellular development and function [32], whereas excessive ROS generation is implicated in myocardial hypertrophy and heart failure [33]. Some studies have reported that ROS are involved in the apoptotic mechanism of MPP^+^-mediated neurotoxicity [34]. As mentioned previously, data from this study showed that treatment with MPP^+^ results in a significant increase in ROS, while pretreatment with ATX and DPI significantly suppressed ROS-generation in PC12 cells in a concentration-dependent manner compared with both the control and the MPP^+^-induced oxidative injury groups (Figure 3). We found that ATX exhibited a significant protective effect against MPP^+^-induced toxicity with no/little toxicity to PC12 cells. In addition, ATX protected neuronal cells against oxidative damage [35].

Findings from a previous study showed that ATX induced levels of HO-1, an antioxidant phase II enzyme, and NRF2 *in vitro*. ATX increased the nuclear levels of NRF2 and the associated protective enzymes HO-1 and NQO-1 in rat liver, and it was demonstrated that the protective properties of ATX are mediated by the NRF2–ARE pathway [36]. In addition, ATX induced HO-1 protein expression in SH-SY5Y cells, protecting against Aβ25–35-mediated cytotoxicity [24]. In keeping with these findings, our study showed that ATX, a powerful antioxidant, significantly increased NRF2 and HO-1 protein and mRNA expression when cells were exposed to MPP^+^. In addition, pretreatment with ATX significantly suppressed NOX2 protein and mRNA expression compared with the MPP^+^ group; this suppression was attenuated by SnPPIX treatment (Figure 6, 7). The results indicate that ATX increased HO-1 and NFR2 protein expression and decreased NOX2 expression, protecting against MPP^+^-induced cytotoxicity in PC12 cells. NOX2 expression was inhibited by ATX and DPI, and over-expression of HO-1 decreased NOX2 expression. We found that NOX2 levels were increased in PC12 cells following MPP^+^ administration and attenuated by ATX pretreatment, which induced HO-1 expression. However, when PC12 cells were cultured in normal growth conditions, ATX did not up-regulate HO-1 and NFR2 expression. The neuroprotective effects of ATX have been reported in several studies using other experimental models [37-39]. Moreover, dietary ATX has been shown to regulate immune responses, oxidative damage and inflammation in humans [40].

## Conclusions

Our results show that ATX protects against MPP^+^-induced oxidative stress via the HO-1/NOX2 axis in a cell model of PD. Our findings suggest that the molecular mechanisms responsible for ATX’s neuroprotection are the suppression of ROS generation, the induction of HO-1 levels and the inhibition of NOX2 expression. Taken together, our data suggest that ATX is a viable neuroprotectant and a potential adjuvant therapy for patients with Parkinson’s disease.

## Abbreviations

ATX: Astaxanthin; MMP^+^: 1-methyl-4-phenyl-pyridine; NOX2: Nicotinamide adenine dinucleotide phosphate oxidase 2; HO-1: Heme oxygenase-1; Nrf2: Nuclear factor erythroid 2-related factor 2; ROS: Reactive oxygen species; PD: Parkinson’s disease; DPI: Diphenyleneiodonium chloride; Hemin: Ferriprotoporphyrin IX chloride; SnPPIX: tin protoporphyrin IX dichloride.

## Competing interests

The authors declare that they have no competing interests.

## Authors’ contributions

QY conceived and supervised the study. BH participated in the DCFH-DA assay, immunohistochemistry, and western blotting, and helped to draft the manuscript. XZ also helped to draft the manuscript. YZ and XC also conceived the study. All authors read and approved the final manuscript.
